# Recent Advances in the Development of Metal/Metal Oxide Nanoparticle and Antibiotic Conjugates (MNP–Antibiotics) to Address Antibiotic Resistance: Review and Perspective

**DOI:** 10.3390/ijms25168915

**Published:** 2024-08-16

**Authors:** Abdullah, Tayyaba Jamil, Muhammad Atif, Shumaila Khalid, Kamel Metwally, Galal Yahya, Mihaela Moisa, Daniela Simona Cavalu

**Affiliations:** 1 Department of Physical Chemistry and Technology of Polymers, Silesian University of Technology, 44-100 Gliwice, Poland; 2 Joint Doctoral School, Silesian University of Technology, 44-100 Gliwice, Poland; tayyaba.jamil@pols.pl; 3 Department of Management Sciences, Silesian University of Technology, 41-800 Zabrze, Poland; 4 Department of Microbiology, Abdul Wali Khan University, Mardan 23000, Pakistan; m.atifjarr2004@gmail.com; 5 Department of Physics, Government Postgraduate, Charsadda 24460, Pakistan; shumailakhalid5050@gmail.com; 6 Department of Pharmaceutical Chemistry, Faculty of Pharmacy, University of Tabuk, Tabuk 71491, Saudi Arabia; kametwally@ut.edu.sa; 7 Department of Microbiology and Immunology, Faculty of Pharmacy, Zagazig University, Al Sharqia 44519, Egypt; galalyehia@zu.edu.eg; 8 Faculty of Medicine and Pharmacy, University of Oradea, P-ta 1 Decembrie 10, 410073 Oradea, Romania; daniela.cavalu@didactic.uoradea.ro

**Keywords:** antimicrobial resistance, antimicrobials, nanoparticles, conjugates, antibiotics, synergism

## Abstract

As per the World Health Organization (WHO), antimicrobial resistance (AMR) is a natural phenomenon whereby microbes develop or acquire genes that render them resistant. The rapid emergence and spread of this phenomenon can be attributed to human activity specifically, the improper and excessive use of antimicrobials for the treatment, prevention, or control of infections in humans, animals, and plants. As a result of this factor, many antibiotics have reduced effectiveness against microbes or may not work fully. Thus, there is a pressing need for the development of new antimicrobial agents in order to counteract antimicrobial resistance. Metallic nanoparticles (MNPs) are well known for their broad antimicrobial properties. Consequently, the use of MNPs with current antibiotics holds significant implications. MNPs, including silver nanoparticles (AgNPS), zinc oxide nanoparticles (ZnONPs), copper nanoparticles (CuNPs), and gold nanoparticles (AuNPs), have been extensively studied in conjunction with antibiotics. However, their mechanism of action is still not completely understood. The interaction between these MNPs and antibiotics can be either synergistic, additive, or antagonistic. The synergistic effect is crucial as it represents the desired outcome that researchers aim for and can be advantageous for the advancement of new antimicrobial agents. This article provides a concise and academic description of the recent advancements in MNP and antibiotic conjugates, including their mechanism of action. It also highlights their possible use in the biomedical field and major challenges associated with the use of MNP–antibiotic conjugates in clinical practice.

## 1. Introduction

**Figure 1:**
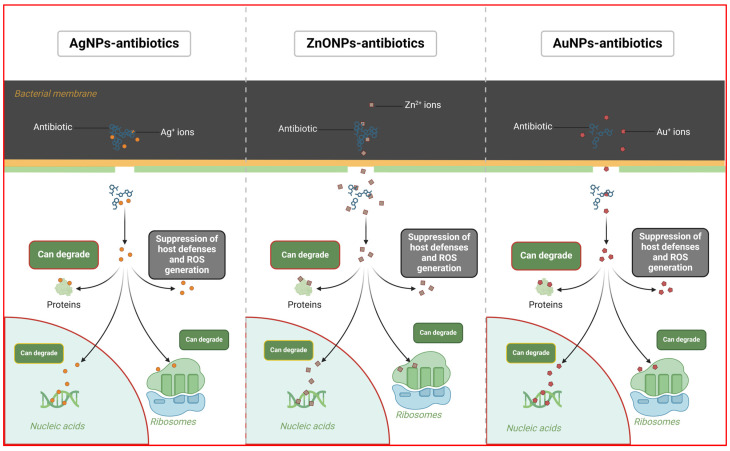
Schematic representation of MNP–antibiotic conjugates and their mechanism of action. Created with BioRender.com.

A major threat to public health is the increasing prevalence of bacterial strains that are resistant to antibiotics [1]. This threat or issue has been worsened by the lowering efficacy of formerly potent medications for the treatment of these infections. New antimicrobial agents are being developed and new means of analyzing them are being explored in an effort to tackle this issue [2,3,4,5]. When multiple drugs are combined, their effectiveness can be enhanced, surpassing the efficacy of each drug on its own. The effectiveness of MNPs can be significantly enhanced by combining them with other antibacterial agents [6,7]. Metal nanoparticles, also known as MNPs, have been the focus of a wide range of studies due to the numerous advantageous characteristics that they possess. These characteristics include their diminutive size, high surface–volume ratio, versatility, increased solubility, surface adaptation, and cellular-level effectiveness. Silver, zinc oxide, copper, gold, nickel, and iron are just a few of the nanoparticles whose antibacterial capabilities have been the subject of much investigation [8,9]. Thus, antibiotics may be more effective when combined with metal nanoparticles (MNPs). Various medical illnesses, including AIDS, cancer, cardiovascular diseases, and microbiological infections, have been effectively treated with pharmaceutical combinations of such drugs [10,11]. This method is commonly employed in clinical settings. The following are a few advantages that can be associated with MNP-antibiotic conjugates: the ability to target multiple microbes and their mechanisms of action, to prevent the rise of resistant microbes, to enable precise intracellular targeting, to prolong the circulation and stability of drugs in the body, to reduce individual dosages to minimize host toxicity, and to broaden the scope of antimicrobial use during therapy [8,12,13]. However, the utilization of MNPs in clinical studies is still limited, as only a few have obtained approval for human use from regulatory agencies like the FDA and EMEA [14]. Regulatory frameworks are essential in addressing the complex nature of nanotechnology, including the risks posed by nanoparticles, such as toxicity and potential exposure and their consequences, as well as the different delivery methods. The approved pharmaceuticals have multiple applications, including serving as substitutes for bone grafts, combating bacterial infections, replenishing the iron levels in the body, and addressing cancer [15]. In the current era, the synergistic effects of MNPs and commercially available antimicrobial drugs have been the subject of vast research, and the quantity of publications has increased dramatically throughout the last five years. There is an ongoing and unresolved debate regarding the efficacy of utilizing antibiotics in conjunction with nanoparticles for the treatment of diseases, particularly those caused by bacteria that exhibit resistance to multiple drugs. To broaden the knowledge of researchers in this particular area, this article presents a concise summary of the current status of MNP–antibiotic conjugates and their mechanism and efficacy in addressing multidrug-resistant bacteria, as well as providing an in-depth examination of the possible uses of MNP–antibiotic conjugates in nanomedicine. Figure 1 presents the general conceptualization of MNP–antibiotic conjugates and their mechanism of action.

## 2. Nanoparticles in Combination with Antibiotics

Various nanoparticles have been extensively researched for the synthesis of MNP–antibiotic conjugates. However, the current studies focus mainly on AgNPs, ZnONPs, CuNPs, and AuNPs in antibiotic conjugates. These nanoparticles possess distinct physicochemical properties, exhibit minimal harm to normal mammalian cells, and demonstrate potent antibacterial properties through multiple mechanisms of action. Therefore, in this article, we focus on these MNPs and their conjugates with antibiotics.

### 2.1. AgNP–Antibiotic Conjugates

Silver and its compounds have recently been the subject of much study regarding their antibacterial properties. Research has shown that relatively small amounts of silver are not hazardous to human cells. Metallic silver’s catalytic oxidation in response to dissolved monovalent silver ions is thought to be the source of silver nanoparticles’ possible antibacterial impact. Compared to conventional and narrow-spectrum antibiotics, microbial resistance to silver is less likely to arise. This is due to the fact that silver hits several biological targets at once and mediates a cascade of changes in the mechanisms that enable microorganisms to build resistance [16,17]. As a result, numerous researchers have directed their attention towards the synthesis of antibiotic–silver nanomaterial conjugates.

Khurana et al. examined the antibacterial properties of AgNPs and their combinations with antibiotics such as tetracycline and kanamycin. They tested these conjugates against *B. subtilis* and *P. fluorescens*, which are microbes involved in biorecycling. The antimicrobial activity of tetracycline showed a significant improvement when combined with silver nanoparticles, with increases ranging from 286% to 346%. The improvement for silver nanoparticles ranged from 154% to 289% for kanamycin, and the synergy between the silver nanoparticles and antibiotics was evident, even at their lowest active concentration of 100 ppm. This research emphasizes the possible impacts linked to the use of antibiotics in conjunction with metal nanoparticles. It implies that these drugs, when used together, can be more harmful to bacteria than when used alone [18]. The research carried out by McShan et al. showed that tetracycline–AgNPs and neomycin–AgNPs, when combined, had a multiplicative impact in terms of *S. typhimurium* growth inhibition. The IC50 values for the tetracycline–AgNPs and neomycin–AgNPs were determined to be 0.07 μg/mL and 0.43 μg/mL, respectively [19]. Antibiotics such as azithromycin, cefotaxime, cefuroxime, fosfomycin, and chloramphenicol were combined with AgNPs by Abo-Shama et al., showing that the synergistic impact against *E. coli* was much stronger than when the antibiotics were used alone. In the presence of AgNPs, all antibiotics exhibited a synergistic effect against *Salmonella* spp. as well. Using oxacillin and neomycin antibiotics together significantly increased the efficacy of the AgNPs against *S. aureus*, in comparison to the antibiotics alone [20].

**Figure 2:**
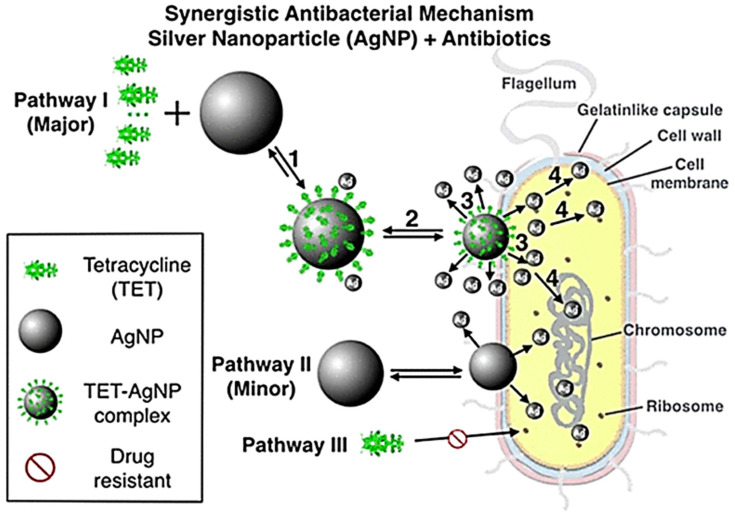
The mechanism of action of AgNP–antibiotic conjugates. Adapted with permission from Ref. [21]. Copyright © 2016, American Chemical Society.

In a study by Deng et al., it was found that the combination of AgNPs with enoxacin, kanamycin, neomycin, and tetracycline exhibited a synergistic effect in inhibiting the growth of *Salmonella* bacteria. Interestingly, ampicillin and penicillin did not show the same level of effectiveness. No specific mechanism was provided, although it was associated with the release of Ag+ ions. Experiments were carried out and both the binding and release of Ag+ from the AgNP–tetracycline conjugate against *Salmonella* were enhanced by 21% and 26%, respectively. However, neither the binding nor the release of Ag+ was affected by penicillin. It was shown that the complex first formed when the AgNPs bound to tetracycline. There was an increase in the release of Ag+ ions inside the *Salmonella* cells as a consequence of the complex produced by tetracycline and the AgNPs. Because of this, a high concentration of Ag+ ions were briefly generated close to the bacterial cell wall, which successfully inhibited the development of the bacteria [21]. This suggests that the release of Ag+ from AgNPs might be the primary factor contributing to the effectiveness of AgNP–antibiotic conjugates, as shown in Figure 2.

The research carried out by Smekalova et al. revealed that when AgNPs were combined with gentamicin and penicillin, they exhibited synergistic effects against *Actinobacillus pleuropneumoniae*. The effectiveness of amoxicillin, gentamicin, and colistin against *A. pleuropneumoniae* and *Pasteurella multocida* was enhanced when combined with AgNPs, despite their initial resistance to these antibiotics. This research shows that silver nanoparticles (AgNPs) have promise as adjuvants for the treatment of bacterial infections in animals [22]. One possible way to deal with bacteria that are resistant to antibiotics is to use certain antibiotics like ampicillin, amoxicillin, or piperacillin together with bacterial β-lactamase inhibitors like clavulanic acid, sulbactam, or tazobactam, as well as silver nanoparticles [23,24,25]. Gupta et al. looked at the antibacterial effects of chitosan-coated AgNPs [26]. The antimicrobial combination demonstrated synergistic effects against *S. aureus*. The MICs of the composites exhibited a significant decrease, approximately ten times lower, compared to the MICs of the AgNPs and chitosan when used individually. Myramistine, another antibacterial agent, showed significantly enhanced effectiveness against *E. coli*, resulting in a 20-fold increase in activity [27]. The treatment of biofilms formed by clinical wound isolates of *P. aeruginosa* and methicillin-resistant *S. aureus* has been reported to be eliminated by a lactoferrin/xylitol hydrogel in conjunction with silver-based wound dressings [28]. An assessment of the synergy was conducted by combining AgNPs with the membrane-permeabilizing antimicrobial peptides polymyxin B and gramicidin S, resulting in observed effects against various Gram-negative bacteria. This evaluation was based on the fractional inhibitory concentration (FIC) index [29].

Multiple studies have suggested that AgNPs can enhance the antibacterial effects of compounds, either through additive or synergistic mechanisms. The studies conducted by Birla et al. and Fayaz et al. demonstrated an additive effect [30,31]. Only a small number of studies have been conducted up to this point that have established the combination action of antibiotics and AgNPs at doses that are lower than those that demonstrate their individual efficacy (i.e., below their MICs) [32,33]. According to the research by Brown et al., ampicillin-functionalized AgNPs had a synergistic impact on many strains of *P. aeruginosa*, *E. aerogenes*, and methicillin-resistant *S. aureus*, which were resistant to antibiotics [34]. Thus, these results suggest that a combination of antibiotics and other multi-level antimicrobials could be a viable option. Together, they may have a stronger antibacterial impact than each antibiotic alone, allowing for the effective elimination or treatment of bacterial infections at lower dosages. In their study on *A. baumannii*, Wan et al. found that tigecycline had an additive impact, while polymyxin B and rifampicin had a synergistic effect when paired with AgNPs [35]. Naqvi et al. conducted a study where they synthesized AgNPs and examined their conjugates with ciprofloxacin, imipenem, gentamycin, and vancomycin. They observed that the combined effect led to a significant increase in antibacterial activity, ranging from 0.2 to 7.0 times (average of 2.8 times) the original value [32]. In a study conducted by Lopez-Carrizales et al., the researchers observed various interactions between AgNPs and ampicillin when tested against 12 different microorganisms. The findings revealed one case of synergy, seven cases of partial synergy, and four instances of additive effects. Similarly, when amikacin was combined with AgNPs, the results showed three cases of synergy, eight cases of partial synergy, and one case of additive effects [36]. In another study conducted by Vazquez-Muñoz et al., it was found that combining AgNPs and kanamycin resulted in a synergistic antimicrobial effect, as indicated by the fractional inhibitory concentration index (FICI) being less than 0.5. Additionally, when AgNPs were combined with chloramphenicol, an additive effect was observed, with the FICI ranging from 0.5 to 1. When β-lactam antibiotics were mixed with AgNPs, no notable impact was seen. Flow cytometry and transmission electron microscopy were used to investigate the impact of modest concentrations of AgNPs (6–7 μg mL^−1^) on bacterial cells. Based on these findings, it seems that structural damage and changes in the bacterial membrane potential increased the cell membrane permeability and eventually caused cell death [37]. There seemed to be no indication that the antibiotics and AgNPs interacted chemically. The study conducted by Jyoti et al. found an impressive increase in the inhibition zone of up to 17.8-fold when amoxicillin was combined with AgNPs against *S. marcescens*, providing strong evidence for the synergistic effect of AgNPs. Thus, AgNPs can enhance the effectiveness of antibiotics [38]. Recent research on AgNP–antibiotic conjugates is presented in Table 1.

### 2.2. Mechanism of Action of AgNP–Antibiotic Conjugates

**Figure 3:**
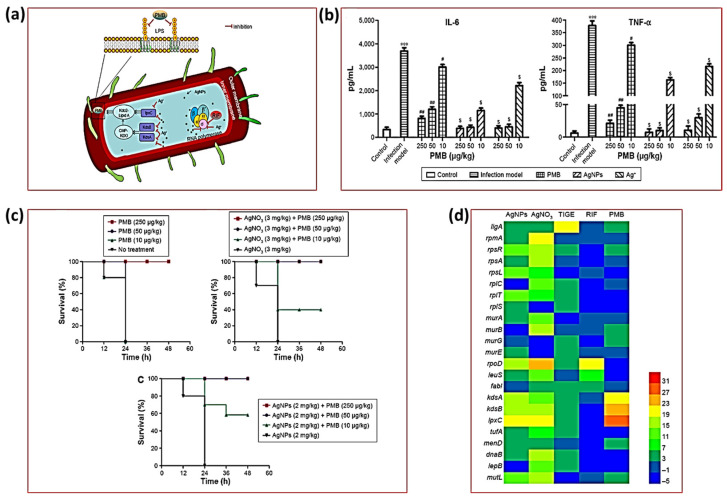
The proposed mechanisms involved in combining AgNPs/Ag+ with antibiotics to combat Gram-negative bacteria. The presence of AgNPs/Ag+ can amplify the membrane lipid damage caused by PMB. Upon entering the cytosol, AgNPs/Ag+ and RIF have the potential to bind intracellular proteins and RNA polymerase (**a**). The release of Ag+ ions from AgNPs has been shown to decrease the levels of proinflammatory cytokines and can reduce the likelihood of *A. baumannii*-mediated infections (**b**). The survival of mice increased when PMB was combined with AgNPs, hence proving their synergism (**c**). A heat map analysis of the sensitivity of *E. coli* (gene-silenced) to AgNPs and antibiotics; the red unit showed the most sensitivity while the blue showed almost equal sensitivity to the control (**d**). All figures adapted from Ref. [35], this work is licensed under the terms and conditions of the Creative Commons Attribution (CC BY license 4.0). The license can be viewed from (https://creativecommons.org/licenses/by/4.0/).

The interaction between AgNPs and antibiotics can result in three different outcomes: synergistic, additive, or antagonistic. The effectiveness of antibiotics and AgNPs against various microbes depends on their respective modes of action. Several studies have proposed various mechanisms of action for these conjugates. The molecular basis of AgNP–antibiotic conjugates was studied in the work by Wan et al. In order to comprehend the combined action of antibiotics and AgNPs, they used *E. coli* cells targeted by antisense RNA [35]. It is probable that the role of polymyxin B (PMB) is responsible for the observed synergy between AgNPs and PMB. PMB increases the penetration of Ag^2+^ ions by increasing the permeabilization of the outer cell membrane and thus facilitates the displacement of Mg^2+^ or Ca^2+^ ions. As a result of this contact, the bacterial cell walls are structurally altered. Another point is that Lipid A of LPS is involved in both PMB and AgNPs’ prospective contributions, suggesting convergence in favor of their synergistic functions. Additionally, this study indicates that the silencing of the LpxC gene is involved in the sensitivity towards AgNPs and AgNO_3_. In LPS biosynthesis, the role of 2-keto-3-deoxyoctanoic acid (KDO) is crucial [41]. The kdsA gene is responsible for encoding the protein KDO-8-phosphate synthetase. Catalyzing the first stage of the KDO synthesis process is a critical function of this protein. The kdsB gene is responsible for encoding CMP-KDO synthetase, a crucial component of the LPS biosynthesis pathway. As a result of silencing these genes, the bacteria became more sensitive to AgNPs, AgNO_3_, and PMB. Suppressing the expression of rpoA, rpoB, rpoC, and rpoD enhanced the susceptibility of *E. coli* to the AgNP–rifampicin conjugate, which provided evidence for their combined therapeutic synergistic effects, as shown in Figure 3a–d.

In addition, previous studies have indicated that AgNPs have the ability to influence the immune responses of host macrophages towards *A. baumannii* and *M. tuberculosis* [35,42]. Accordingly, *A. baumannii* not only increased Ca^2+^ ions’ influx but also released TNF-α and IL-6. The levels of TNF-α and IL-6 in the plasma of mice treated with AgNPs were found to be significantly lower in this research. These findings suggest that inhibiting proinflammatory signals could potentially provide protection against *A. baumannii* infections.

In addition to the molecular basis, various theories have been put forth to comprehend the mechanism of action of AgNPs and their combination with antibiotics. According to a theory, AgNPs have the potential to interfere with the cellular respiratory chain by reacting with oxygen. Furthermore, the interaction between AgNPs and the cell membrane ultimately results in cell death. According to a theory put forward by [43], AgNPs may have the ability to inhibit the unwinding of DNA, thereby exerting antibacterial effects. Another theory suggests that reactive oxygen species (ROS) are responsible for the oxidative damage that AgNPs exhibit; these ROS are thought to contribute to the antibacterial action of AgNPs [44]. The interaction between AgNPs and bacteria is not easily explained by a single, specific mechanism, unlike for antibiotics. The study described in [45] found that AgNPs had detrimental effects on the bacterial cell wall, leading to changes in membrane permeability and the collapse of plasma membrane potency. In addition, AgNPs have been found to have various effects on biological systems, including interactions with DNA, enzyme inactivation, influences on metabolic processes, alterations of protein expression, and damage to the respiratory chain [46]. Silver ions are released from the surfaces of their nanoparticles and then enter the cell of the bacterium. After penetration, these ions produce ROS, which are capable of destroying biomacromolecules [47]. There are number of ways in which AgNPs improve the antibiotic–cell interaction. For example, by modifying the membrane permeability, they may facilitate antibiotic entrance into the bacterial cell. Another possibility is that the cell wall may be compromised by a combination of antibiotics and AgNPs. AgNPs have the potential to hinder the hydrolytic β-lactamases that bacteria produce, particularly in the case of β-lactam antibiotics. The combined effects of antibiotics and AgNPs can result in cellular damage and weakness, ultimately leading to cell death. In a previous study [23], it was suggested that this combined action might be associated with three factors: the generation of hydroxyl radicals, interference with protective cellular activities, and the possibility of preventing the development of biofilms. Hence, the utilization of antibiotics in conjunction with AgNPs appears to be a more potent approach to augmenting antibiotics’ effectiveness when compared to other adjuncts presently employed in clinical settings. The combination shows promise in reducing superbugs, slowing the rise of antibiotic-resistant microorganisms, and enhancing the synergistic effects of medicines.

**Figure 4:**
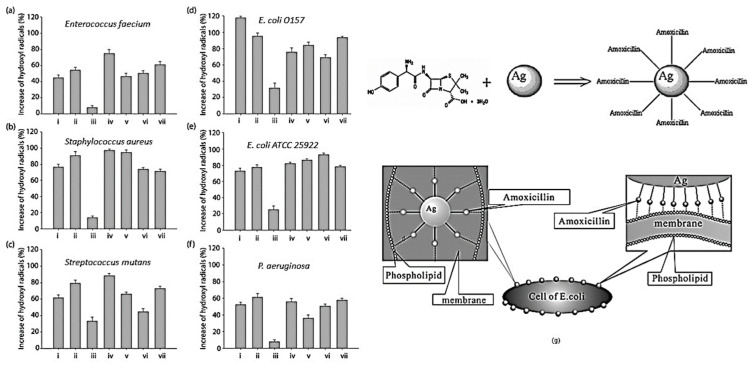
Hydroxyl radical formation assay against tested microorganisms (**a**–**f**), i: AgNPs; ii: ampicillin; iii: chloramphenicol; iv: kanamycin; v: AgNPs–ampicillin; vi: AgNPs–chloramphenicol; vii: AgNPs–kanamycin, adapted with permission from Ref. [23], Copyright © 2012 Microbiology Society. The combination of AgNPs and ampicillin can target the cell membrane of *E. coli* (**g**), adapted with permission from Ref. [52], Copyright © 2005, Institute of Physics Publishing Ltd.

Nevertheless, the European Antimicrobial Resistance Surveillance Network (EARS-Net) [48] has shown an alarming increase in the prevalence of bacteria that are resistant to various antibiotics and other drugs designed to target certain resistance mechanisms. Instead of depending on the effects of conventional antibiotics, a new antimicrobial medication that aims to prevent the development of bacterial resistance must instead target many cellular levels. It is well known that silver, in its many forms (metal nanoparticles, compounds, etc.), has a profound effect on many bacterial metabolic processes and structures. Significant research has focused on how silver nanoparticles (AgNPs) affect microorganisms. Studies have shown that AgNPs can inhibit bacterial enzyme activity, interfere with cell wall and metabolic processes, build up in the cytoplasmic membrane and render it more permeable, lower the plasma membrane potential, interact with DNA, and produce reactive oxygen species, which can harm biomacromolecules [44,45,49]. All pathogenic microbes, even those with remarkable resistance to antibiotics, may be killed or at least slowed down by AgNPs because of their multi-level mechanism of action. At concentrations varying from small units to many tens of mg/L, this impact has been seen. Hence, AgNPs can be regarded as a viable option for pairing with antibiotics. Hwang et al. conducted a hydroxyl radical formation assay to explore the synergism between AgNPs and antibiotics. Their findings suggest that AgNPs play a role in the formation of OH radicals, which could be a crucial factor in the synergistic effects of AgNP–antibiotic conjugates [23]. Figure 4a–g demonstrates that when combined with chloramphenicol, AgNPs exhibited the greater production of OH radicals against the tested microorganisms. However, the reported results in this study were not consistent for all antibiotics combined with AgNPs. At this time, there is a lack of published information about the development of bacterial resistance to AgNPs or the deactivation of AgNPs’ antibacterial effects [50]. However, bacteria can be resistant to ionic silver. Bacteria may develop resistance by reducing Ag+ to a less harmful oxidation state; it is likely that the mechanism of Ag+ resistance involves actively removing Ag+ from the cell through either P-type ATPases or chemiosmotic Ag+/H+ antiporters [26,51]. This particular type of resistance, however, has not yet been observed.

### 2.3. ZnONP–Antibiotic Conjugates

The US Food and Drug Administration (FDA) has determined that zinc oxide nanoparticles (ZnO NPs) are useful for consumption [53]. There is evidence from previous studies that ZnONPs have antibacterial properties against a wide range of microorganisms [54]. It should be noted that these nanoparticles have the remarkable ability to target bacteria selectively, with very little damage to human cells [54]. Thus, it is proposed that ZnONPs, when used with antibiotics, might boost the efficacy of these commercial drugs.

**Figure 5:**
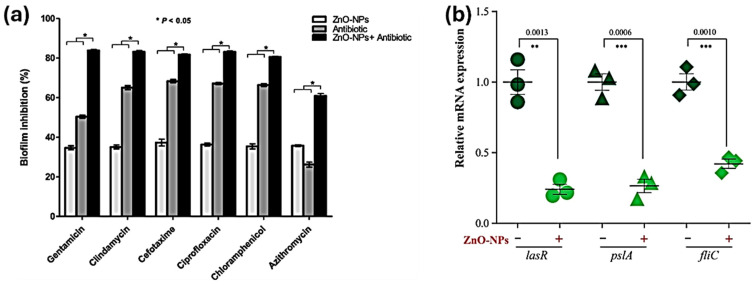
Enhanced biofilm reduction and inhibition of *S. aureus* when combining ZnONPs with antibiotics, * *p* ≤ 0.05 (**a**), adapted from Ref. [65], this work is licensed under the terms and conditions of the Creative Commons Attribution (CC BY license 4.0). The license can be viewed from (https://creativecommons.org/licenses/by/4.0/). The effect of ZnONPs on the expression of *lasR*, *fliC*, and *pslA* genes, which play a crucial role in biofilm formation. The findings demonstrate the relative mRNA expression of each gene in relation to *16S rRNA*, ** *p* ≤ 0.01; *** *p* ≤ 0.001 (**b**), adapted from Ref. [70], this work is licensed under the terms and conditions of the Creative Commons Attribution (CC BY license 4.0). The license can be viewed from (https://creativecommons.org/licenses/by/4.0/).

Recent research by Abo-Shama et al. showed that ZnONPs significantly increased the efficacy of imipenem in combating *K. pneumoniae* and *E. coli* [20]. A study conducted by Sharif et al. found that there was a synergistic effect of levofloxacin against MRSA when combined with ZnONPs. This effect was observed to be dose-dependent [55]. Fadwa et al. examined the effectiveness of combining colistin with ZnONPs against *P. aeruginosa*. The results of the time-kill growth experiment demonstrated a synergistic effect at a minimum inhibitory concentration (MIC) of 2 µg/mL. This combination was able to completely inhibit bacterial growth, resulting in zero colonies after 24 h of incubation [56]. At a dosage of 10 μg/mL, ciprofloxacin-conjugated ZnONPs also showed synergistic antibacterial activity against multidrug-resistant strains of *E. coli*, *S. aureus*, and *Klebsiella* spp. [57]. Adding ZnONPs to ciprofloxacin and ceftazidime has been investigated as a means to enhance its antibacterial efficacy against *A. baumannii* [58]. The incorporation of ZnONPs enhanced the cellular absorption of antibiotics in bacteria. Amoxicillin-loaded ZnONPs have demonstrated noteworthy antibacterial efficacy against both Gram-positive and Gram-negative bacteria causing infections. *S. epidermis* displayed the largest zone of inhibition (MIC) in this study [59]. An effective combination of antibiotics including oxacillin, azithromycin, cefuroxime, cefotaxime, oxytetracycline, and fosfomycin was seen to have a much greater impact against *E. coli* in recent research [20]. ZnONPs coupled with the antibiotics ofloxacin, norfloxacin, and cephalexin showed potential against the pathogens *E. coli*, *S. aureus*, and *P. aeruginosa* [60]. The addition of ZnONPs has been found to enhance the antimicrobial efficacy of beta-lactam antibiotics, including ciprofloxacin, imipenem, ceftazidime, and erythromycin [61,62,63]. A study conducted by Chandrasekaran et al. suggested that the doping of ZnONPs and their combination with chloramphenicol can increase their activity against *S. aureus*, *E. lentum*, *P. vulgaris*, and *E. aerogens* [64]. ZnONP–antibiotics showed a significant reduction of *S. aureus* biofilms, as shown in Figure 5a [65]. Table 2 contains a collection of recent experiments that have been conducted on ZnONP and antibiotic conjugates. Furthermore, the incorporation of ZnONPs with various natural metabolites presents an exciting opportunity for further exploration within the scientific communities. Nanoparticle and drug conjugate development as novel antibacterial agents may benefit greatly from this research [66].

**Table 2:** Recent investigations on conjugates of zinc oxide nanoparticles with antibiotics.

MOs	Antibiotics with Synergistic Effects	Antibiotics with Antagonistic Effects	No Effect	Ref.
*E. coli*	Fosfomycin, Gentamicin, Oxytetracycline, Azithromycin, Oxacillin, Cefuroxime, Cefotaxime	Ampicillin/Sulbactam	Neomycin, Chloramphenicol	[20]
*S. aureus*	Chloramphenicol, Azithromycin, Cefotaxime, Oxytetracycline, Cefuroxime, and Fosfomycin	Oxacillin, Ampicillin/Sulbactam, and Neomycin	Gentamicin	
*Salmonella* spp.	Cefuroxime, Fosfomycin, and Oxacillin	Oxytetracycline, Neomycin Gentamicin, Cefotaxime, Azithromycin, Ampicillin/Sulbactam, and Chloramphenicol		
*P. aeruginosa*	Colistin			[56]
*S. aureus*	Levofloxacin			[55]
*H. pylori*	Amoxicillin			[67]
*A. baumannii*	Colistin			[68]
*S. aureus*, *E. lentum*, *P. vulgaris*, and *E. aerogens*	Chloramphenicol			[64]
*A.* *baumannii*	Ciprofloxacin			[69]
*P. aeruginosa*	Meropenem			[70]

### 2.4. Mechanism of Action of ZnONP–Antibiotic Conjugates

It is still not completely known how ZnONPs exert their antibacterial properties in combination with antibiotics. However, prior studies have demonstrated the bactericidal and bacteriostatic effects of hydrogen peroxide (H_2_O_2_). This occurs as a result of the formation of reactive oxygen species (ROS) during the process of reaction. It is believed that ZnONPs are responsible for the cellular damage that they produce because of their capacity to rupture cell walls and membranes. This allows them to enter cells more easily by limiting the extent of proton stimulation that they receive.

As a consequence of electrostatic induction between the bacterial cell membrane and nanoparticles, nanostructures that are composed of transition metal oxides display antibacterial characteristics. The nanoparticles remain on the bacterial surface for an extended duration without traversing the cell wall. As a result of their capacity to alter the viscosity of the bacterial membrane and to impair the performance of certain ionic pumps, nanoparticles (NPs) have the potential to disturb the growth of bacteria. In the end, this has an effect on the transportation interactions between the bacterial cell and the fluid. The synergistic effects of ZnONPs were attributed to oxidative stress. Bacterial cells can uptake Zn^2+^ ions, which then interact with respiratory enzymes in the cell membrane, hindering their functionality. Oxidative stress plays a crucial role in boosting antibacterial activity. Reactive oxygen species (ROS) generated within living organisms have the potential to induce oxidative stress, leading to irreversible damage to bacterial cells. Bacteria can be eliminated and broken down by targeting biological components like nucleic acids, proteins, polysaccharides, and lipids. The particles exhibit an enhanced adsorption capacity, decreased band gap energy, and an increased surface area, enabling this phenomenon. Additionally, these substances can lead to the hydrolysis and fragmentation of DNA [67]. ZnONP–ciprofloxacin’s mechanism of action has often been studied via the use of a cell membrane damage assay [57]. El-Telbany et al. [70] explained the molecular mechanism of ZnONPs and their conjugates with meropenem against *P. aeruginosa*. They described that ZnONPs can suppress the expression of genes (fliC, pslA, and lasR), as shown in Figure 5b; these genes are involved in biofilm formation in *P. aeruginosa*. These results suggest that meropenem and ZnONPs can act synergistically as meropenem targets the penicillin-binding proteins [71] while ZnONPs can repress the expression of viable genes responsible for the growth of *P. aeruginosa*.

### 2.5. Cu/CuONP–Antibiotic Conjugates

**Figure 6:**
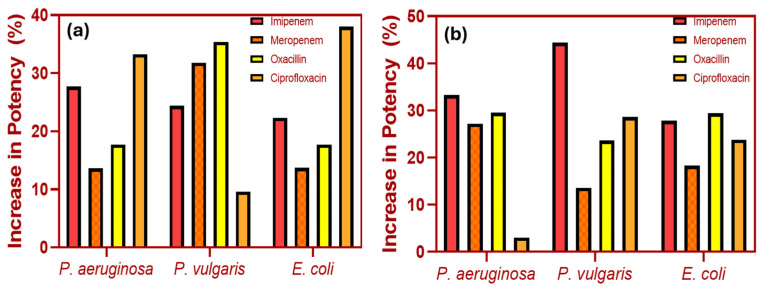
The increase in the potency of commercial antibiotics when combining them with CuNPs (**a**) and Cu-Ni NPs (**b**), the data is compiled from Ref. [74], this work is licensed under the terms and conditions of the Creative Commons Attribution (CC BY license 4.0). The license can be viewed from (https://creativecommons.org/licenses/by/4.0/).

There have been investigations on the potential synergy between antibiotics and copper and copper-based nanoparticles in the treatment of diverse bacterial strains. For example, in their study of *P. fluorescens* and *B. subtilis*. Khurana et al. looked at the synergistic impacts of tetracycline, kanamycin, and copper nanoparticles [18]. Yaqub et al. studied the combination of Cu and Cu-based nanoparticles with doxycycline against *E. coli* and *P. aeruginosa* [72]. Moreover, Naqvi et al. investigated the antimicrobial activity of copper and copper-based nanoparticles in conjunction with ciprofloxacin and streptomycin against *E. coli*, *K. pneumoniae*, *P. aeruginosa*, *P. mirabilis*, and *O. oxytoca* [73]. Shah et al. showed that the combination of CuNPs and copper-based NPs with imipenem, meropenem, and ciprofloxacin could enhance their activity up to 0.5–3-fold [74], as shown in Figure 6a,b. These studies suggest that the observed synergism could be attributed to the release of Cu^2+^ ions from the complexes, which can cause harm to bacterial cells. According to Vasiliev et al., the binding of CuNPs with antibiotics might be lower compared to AgNPs [75]. There is limited research on the molecular antibacterial mechanisms of CuONPs; however, some potential mechanisms are discussed below.

### 2.6. Mechanism of Action of Cu/CuONP–Antibiotic Conjugates

**Figure 7:**
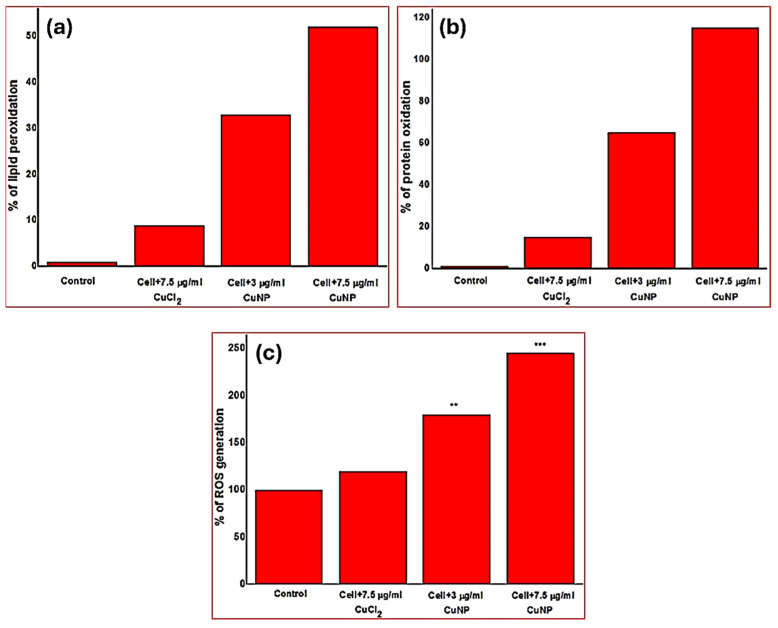
The antibacterial mechanism of CuNPs against *E. coli* by degrading its membrane lipids (**a**) and proteins (**b**) and by the production of ROS ∗∗∗ = *p* < 0.001 and ∗∗ = *p* > 0.01 (**c**), adapted with permission from Ref. [78], Copyright © 2014, Institute of Physics Publishing Ltd.

Cu and CuO nanoparticles have a variety of antibacterial mechanisms that consist of both physical and chemical interactions with bacterial cells. Cu-based NPs can generate reactive oxygen species (ROS), hydroxyl radicals, superoxide anions, and hydrogen peroxide; these radicals induce oxidative stress in bacterial cells, leading to the impairment of cellular components such as DNA, proteins, and lipids, which ultimately results in the death of cells [74]. Furthermore, Cu and CuONPs release Cu²⁺ ions into the surrounding environment. These ions have the ability to pass through bacterial cell membranes and interact with targets inside the cells. Cu²⁺ ions have the ability to bind to proteins and enzymes that are crucial for bacterial metabolism and DNA replication, causing disruption. The combination of these mechanisms with antibiotics often enhances their effectiveness [18,72,73,74]. In a study conducted by Heinlaan et al., it was demonstrated that the toxicity of CuONPs to both *Vibrio fischeri* bacteria and crustaceans was attributed to solubilized Cu^2+^ ions [76]. The antibacterial mechanisms of CuONPs were further investigated through the use of recombinant *E. coli* [77]. The presence of CuO nanoparticles, particularly those with a positive charge, resulted in notable levels of lipid peroxidation, protein oxidation, and reactive oxygen species (ROS) formation [78], as shown in Figure 7a–c. The modification of the CuNPs’ surface charge through surface functionalization is crucial in the bacterial inactivation process. Due to the negative charge of the bacteria’s cell wall, the positively charged CuNPs have a stronger affinity for the bacterial cell walls, resulting in the more effective inactivation of bacteria [78]. Additional research is advised to assess the potential synergistic mechanism of copper nanoparticles when used alongside antibiotics. It is proposed that Cu-based NPs, when combined with antibiotics, could target multiples sites in bacteria and lead to synergistic effects. However, the activity may depend on various factors that can affect the antibacterial activity of Cu and CuONPs, such as the particle size, shape, concentration, and pH. Higher concentrations of nanoparticles tend to boost the antibacterial activity, but they can also heighten the toxicity to human cells.

### 2.7. AuNP–Antibiotic Conjugates

Gold nanoparticles are widely recognized for their antimicrobial properties and have gained significant interest as a potential substitute for traditional antibiotics because of their unique antibacterial characteristics. The use of antibiotics in conjunction with gold nanoparticles shows great potential in addressing the issue of antimicrobial resistance. The improved antibacterial activity of a gold nanocarrier loaded with amoxicillin against *S. pneumoniae*, *E. coli*, and *S. aureus* was shown by Perez et al. [79]. Thus, gold nanosystems could potentially serve as a viable treatment option to address antibiotic resistance, while also minimizing the adverse effects associated with high antibiotic doses. A study was conducted to enhance the antibacterial effectiveness and drug delivery efficiency against *P. aeruginosa* by loading ceftazidime into a gold nanoparticle [80]. The findings indicated that the ceftazidime-loaded nanoparticles exhibited enhanced efficacy in comparison to the native ceftazidime when targeting *P. aeruginosa*. The standard serial dilution approach was used to evaluate the efficacy of AuNPs in conjunction with low quantities of ampicillin MRSA. It is conceivable that this strategy may provide a workable and permanent solution to the issue of antibiotic resistance [81].

**Figure 8:**
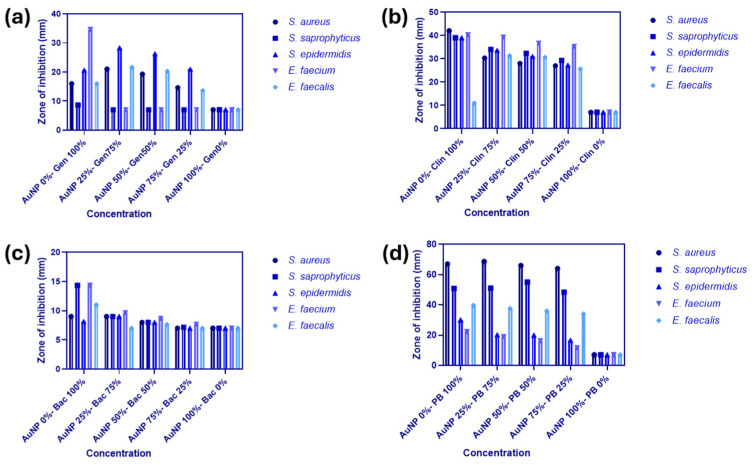
The AuNP–antibiotic conjugates and their synergism: (**a**) gentamicin, (**b**) clindamycin, (**c**) bacitracin, (**d**) polymyxin B. This data is compiled from Ref. [82], this work is licensed under the terms and conditions of the Creative Commons Attribution (CC BY license 4.0). The license can be viewed from (https://creativecommons.org/licenses/by/4.0/).

Figure 8a–d show how five different antibiotics’ antibacterial activity was affected by the addition of colloidal gold nanoparticles in the work of Dadpour and Doust [82]. The antibacterial activity against some Gram-positive cocci was improved when antibiotics and AuNPs were mixed at a ratio of 25:75, as compared to antibiotics alone. Instead of just combining the antibiotic-coated AuNPs, it is advised to construct stable conjugates for more noticeable results. Researchers may benefit from further data on the in vivo effects and toxicity of AuNPs if they wish to draw stronger conclusions [82].

### 2.8. Mechanism of Action of AuNP–Antibiotic Conjugates

**Figure 9:**
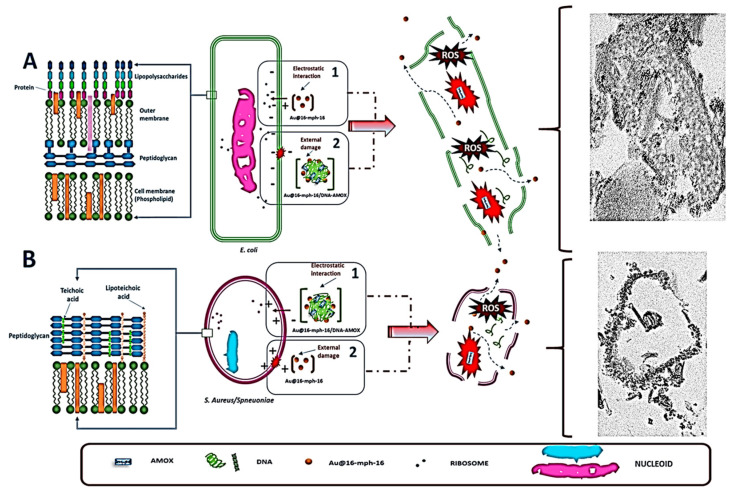
Illustration of the antibacterial mechanism of amoxicillin-loaded AuNPs against Gram-positive and Gram-negative bacteria: (**A**) *E. coli*, (**B**) *S. aureus*. On the left side of the image the images show differences in the cell walls of Gram-negative and Gram-positive bacteria repectively, adapted from Ref. [79], this work is licensed under the terms and conditions of the Creative Commons Attribution (CC BY license 4.0). The license can be viewed from (https://creativecommons.org/licenses/by/4.0/).

The antibacterial properties of gold nanoparticles (AuNPs) are influenced by various significant factors. AuNPs, due to their nanoscale size, have the ability to penetrate the bacterial cell membrane. This penetration has the potential to disrupt the membrane and release cellular components. The proteins, lipids, and nucleic acids found in bacterial cells are vulnerable to harm when AuNPs are exposed to light or other stimuli. This is because they may produce free radicals and reactive oxygen species (ROS). In addition, the interaction between AuNPs and preexisting disulfide bridges or thiol groups can lead to the damage of proteins, thereby disrupting cellular molecular systems. It is possible that some AuNPs can hinder bacterial growth by interfering with essential metabolic processes or DNA replication. Cellular respiration is also impeded because they negatively affect the electron transport chain and ATP synthesis, both of which are essential for the energy production of bacteria. By damaging DNA and hence triggering mutations and cell death, nanoparticles may impede normal biological processes. Furthermore, they have the potential to impact gene expression. The deposition of AuNPs on different bacterial cell components, such as the cell wall, plasma membrane, or cytoplasm, can lead to localized injury [79]. Furthermore, these compounds have the ability to modify the structure and function of bacterial proteins via interactions with them. Crucial biomolecules may be oxidized as a consequence of this interaction’s induction of oxidative stress in bacterial cells. It is still not known how exactly AuNPs kill bacteria. It is known, however, that AuNPs interact directly with cell membranes, produce reactive oxygen species (ROS), and damage DNA and proteins by absorbing free gold ions, all of which contribute to oxidative stress, as shown in Figure 9.

## 3. Combination of Other Nanoparticles with Antibiotics

There are other nanoparticles, such as Fe, Pt, Pd, Ba, and Ti, that have the potential to greatly enhance the effectiveness of commercial antibiotics through synergistic effects. Bimetallic nanoparticles have also been combined with antibiotics. A study conducted by Vernaya et al. found that the combination of iron nanoparticles and gentamicin can have a remarkable impact as an antibacterial agent. They found that FeNPs can be modified to specifically target bacterial cells, resulting in the improved delivery and effectiveness of gentamicin. This is significant because gentamicin is known for its ability to inhibit protein synthesis. Observations have shown an increase in antibacterial activity against *P. aeruginosa* and *E. coli* [83]; these nanoparticles were also combined with Cu and similar results were obtained. In another study, Ag–Au bimetallic NPs were combined with doxycycline to treat complicated skin infections. The results showed enhanced activity against *P. aeruginosa*, *E. coli*, *S. aureus*, and *M. luteus* [84]. Eleftheriadou et al. combined bimetallic Cu–Zn NPs with meropenem and observed synergistic activity against *P. aeruginosa* [85]. Similarly, Ag–Pt NPs were combined with ampicillin, streptomycin, rifampicin, etc., and synergistic effects were achieved [86]. Therefore, the desired synergistic effect between antibiotics and nanoparticles can be achieved by combining single, bimetallic, or trimetallic nanoparticles from various sources. However, it is essential to evaluate both the toxicological effects and pharmacological properties.

## 4. Future Perspectives

Combining MNPs with antibiotics has proven to be highly advantageous, especially in addressing the issue of antimicrobial resistance. As mentioned, many studies have examined the use of MNPs in combination with commonly used antimicrobial agents such as antibiotics, antifungals, and antivirals. However, the majority of these studies have not conducted in vivo experiments to assess the cytotoxicity and pharmacokinetics of MNP–antibiotic conjugates. Some metals and metal oxides have been found to have detrimental effects on human cells and microbes and are not easily eliminated or metabolized by the body. It is important to mention that the FDA and EMEA have approved only a limited number of MNPs for clinical use. It is crucial to promptly implement measures that establish and enforce robust standards to effectively address the toxicity of nanoparticles and mitigate their potential negative effects. Understanding the pharmacokinetics of MNPs and conjugates is crucial in improving their selectivity and reducing their accumulation in non-target cells. A restricted selection of materials was analyzed during the development of conjugated agents. Thus, it is necessary to explore additional combinations that require further investigation. The leakage of drugs from nanoparticles can pose a risk to the effectiveness of MNP–antibiotics and potentially contribute to the misuse of antibiotics. Therefore, we suggest that covalently bound methods are more effective compared to simple physical adsorption. Conjugating MNPs with antibiotics poses a significant challenge, as it necessitates thorough characterization to determine the most effective combination. Hence, it is recommended to accurately characterize and combine MNPs with antifungals, antivirals, and antiparasitic agents in order to gain further insights and comprehend the synergistic process. This knowledge will be instrumental in utilizing these materials effectively in future nanomedicine. Additionally, MNP conjugates offer numerous benefits, including reduced medication doses per patient, decreased cytotoxicity, and a wider range of protection against antimicrobial resistance.

## Figures and Tables

**Table 1:** Some promising studies regarding AgNP–antibiotic conjugates.

MOs	Antibiotics with Synergistic Effects	Antibiotics with Antagonistic Effects	No Effect	Ref.
*E. coli*	Cefotaxime, Azithromycin, Oxacillin, Ampicillin/Sulbactam, Cefuroxime, Fosfomycin, and Chloramphenicol	Gentamicin, Neomycin	Oxytetracycline	[20]
*S. aureus*	Chloramphenicol, Azithromycin, Cefotaxime, Gentamicin, Cefuroxime, Ampicillin/Sulbactam, and Cefotaxime	Oxacillin and Neomycin		
*Salmonella* spp.	Neomycin, Azithromycin, Cefotaxime, Gentamicin, Cefuroxime, Fosfomycin, Chloramphenicol, Oxacillin, and Ampicillin/Sulbactam			
*B. subtilis* and *P. fluorescens*	Tetracycline, and Kanamycin			[18]
*S. typhi*	Tetracycline, Enoxacin, Kanamycin, and Neomycin	Penicillin		[21]
*S. uberis*, *S. aureus*, *Actinobacillus pleuropneumoniae*		Ampicillin		[22]
*Actinobacillus pleuropneumoniae*	Penicillin			
*S. aureus*	Gentamicin			
*E. coli*	Colistin			
*E. coli*	Ampicillin, Ampicillin/Sulbactam, Cefazolin, Cefuroxime			[39]
*P. aeruginosa*	Ciprofloxacin, Meropenem, Piperacillin			
*S. aureus*	Gentamicin, Vancomycin, Ciprofloxacin			
*A. baumannii*	Polymyxin B and Rifampicin			[35]
*E. coli*, *K. pneumoniae*, *S. aureus*, *E. faecalis*, *P. aeruginosa*, *Bacillus* spp., *A. baumanii*, and *M. luteus*	Ciprofloxacin, Vancomycin, Gentamycin, Imipenem, and Trimethoprim			[32]
*E. faecium*, *A. baumannii*, *K. pneumoniae*, *E. coli*, *P. aeruginosa*, and *Morganella morganii*	Ampicillin and Amikacin			[36]
*E. coli*, *S. typhimurium*, *S. aureus*, *B. subtilis*	Kanamycin, Chloramphenicol		Biapenem, Aztreonam, Ampicillin	[37]
*B. cereus*, *S. epidermidis*, *S. aureus*, *E. coli*, *S. typhimurium*, *K. pneumoniae*, *B. subtilis*, *S. marcescens*	Cefepime, Amoxicillin, Cefotaxime, Vancomycin, Kanamycin, Vancomycin, Tetracycline, and Streptomycin			[38]
*B. anthracis*, *S. enterica*, *E. coli*, *S. aureus*, *V. parahaemolyticus*, and *B. cereus*	Vancomycin, Lincomycin, Oleandomycin, Novobiocin, Penicillin G, Rifampicin, and Cycloheximide			[40]
